# Reconceptualizing regional anesthesia as a systemic modulator: a hypothesis-driven gut-brain axis perspective

**DOI:** 10.3389/fnins.2026.1785236

**Published:** 2026-03-09

**Authors:** Man Li, Ya-ting Li, Zhi-jun Qin

**Affiliations:** 1 Department of Anesthesiology, Sichuan Province Orthopedic Hospital, Chengdu, China; 2 Department of Anesthesiology, The Second Affiliated Hospital of Inner Mongolia Medical University, Hohhot, China

**Keywords:** gut-brain axis, neuroimmune modulation, perioperative, regional anesthesia, translational medicine

## Abstract

Traditionally, regional anesthesia has been considered a local analgesic technique with benefits limited to the site of blockade. However, emerging preclinical and clinical evidence indicates that it may produce systemic effects by modulating the gut-brain axis. The gut-brain axis is a bidirectional regulatory system that integrates neural, immune, endocrine, and microbial signaling and is crucial for managing perioperative stress, inflammation, and physiological balance. This perspective article examines how regional anesthesia influences the gut-brain axis through several interconnected pathways, including neural modulation, immune regulation, endocrine effects, and indirect alterations in gut microbiota. We argue for reconceptualizing regional anesthesia as a systemic modulator and discuss its potential applications in perioperative recovery and chronic disease management. We conclude by calling for interdisciplinary research and mechanism-focused clinical trials to integrate this perspective into a more holistic model of perioperative medicine.

## 1 Introduction

Regional anesthesia, especially peripheral nerve blocks and neuraxial anesthesia, is a cornerstone of perioperative care primarily due to its potent local analgesic effects (Hutton et al., 2018; Schug et al., 2006). Its mechanism of action has been traditionally understood as the reversible pharmacological blockade of specific nerve pathways by local anesthetics (Taylor and McLeod, 2020). By preventing nociceptive signals from reaching the central nervous system, it provides excellent targeted pain relief, thereby reducing the need for systemic opioids and associated side effects like postoperative nausea and respiratory depression (Bingham et al., 2012). Consequently, this conventional view frames regional anesthesia primarily as a localized intervention, with its benefits considered largely confined to the anesthetized anatomical region (Liu and Wu, 2007).

In addition to this mechanistic and physiological relevance, regional anesthetic techniques offer distinct clinical advantages that have strengthened their role in contemporary practice. Local anesthetics are inherently cost-effective and widely accessible (Graff et al., 2023). Their regional application limits systemic drug exposure, enhancing safety (Hutton et al., 2018). Furthermore, within multimodal analgesic strategies, these techniques substantially decrease the dependency on general anesthetics and opioids, thereby lowering the overall risks of postoperative nausea, respiratory depression, and delirium (Li et al., 2022).

However, accumulating evidence from both clinical and basic research suggests that the impact of regional anesthesia extends well beyond localized neural blockade (Sessler et al., 2019). A growing body of evidence demonstrates that regional anesthesia confers systemic benefits across diverse surgical settings, including major abdominal, thoracic, orthopedic, and oncologic procedures (Reysner et al., 2024). These systemic benefits encompass areas such as immune modulation, attenuation of the neuroendocrine stress response, and enhancement of overall postoperative recovery (Reysner et al., 2024; Grosu and Lavand'homme, 2015). A clinically and mechanistically significant example is the facilitation of gastrointestinal recovery following abdominal surgery (Shi et al., 2014). This specific effect underscores the involvement of sophisticated gut-brain signaling pathways, which are central to maintaining perioperative homeostasis (Costes et al., 2014). This broader impact is further evidenced by documented associations with modulated immune responses and potential effects on long-term outcomes like cancer recurrence and cardiovascular events, all pointing to the involvement of systemic regulatory pathways (Dubowitz et al., 2018; Myles et al., 2011).

In this context, the gut-brain axis—a bidirectional communication network integrating the central nervous, autonomic, enteric, and immune systems along with gut microbiota—has emerged as a critical systemic regulatory hub (Wang et al., 2021). This axis plays a fundamental role in pain perception, stress responses, inflammation, and maintaining visceral homeostasis (Cryan et al., 2019).

Building on this, a compelling hypothesis posits that regional anesthesia may modulate systemic physiology by directly or indirectly influencing the gut-brain axis (Schäper et al., 2013). For example, by attenuating surgical stress and nociceptive signaling, nerve blockade can reduce sympathetic nervous system hyperactivity (Schäper et al., 2013). This sympathetic suppression can, in turn, improve intestinal blood flow, regulate gut barrier function, and influence local and systemic immune activity (Reysner et al., 2024). Furthermore, by blunting the surgical stress and inflammatory response, regional anesthesia may create a systemic milieu that favorably alters gut-brain communication (Schäper et al., 2013; Hahnenkamp et al., 2004). Thus, a local nerve block might initiate a cascade of events through the gut-brain axis, leading to widespread physiological effects.

This perspective article, therefore, argues for a paradigm shift in understanding regional anesthesia: from a focus on local analgesia to its recognition as a modulator of systemic physiology. We aim, first, to explore the evidence linking regional anesthesia to systemic effects mediated via the gut-brain axis. Second, we will synthesize current knowledge into a coherent mechanistic framework explaining this local-to-systemic translation. Finally, we will explore the clinical implications of this paradigm for perioperative strategy design and future research directions. Through this perspective, we seek to provide a deeper mechanistic rationale for its use and highlight its potential as a broader therapeutic tool in perioperative medicine.

## 2 Theoretical basis: gut-brain axis integration in anatomy and physiology

### 2.1 Components of the gut-brain axis

**Figure 1:**
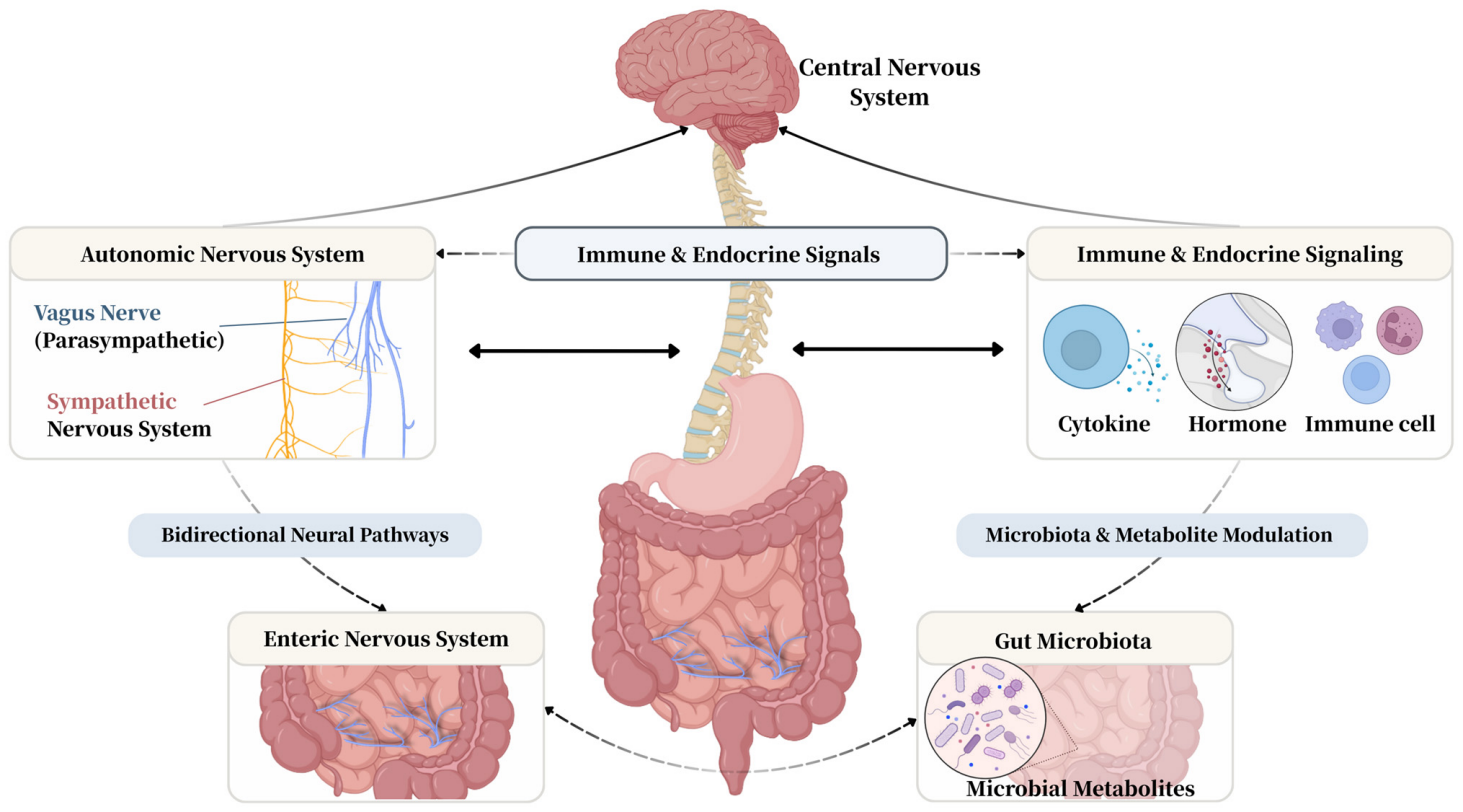
Anatomical and physiological components of the gut-brain and their potential interactions in the perioperative context.

The gut-brain axis is an integrated physiological system comprising multiple, interconnected components, not a single structure (Carabotti et al., 2015). Its core architecture consists of four main elements (Holzer and Farzi, 2014). First, the central nervous system acts as the primary command and integration center (Mayer et al., 2015). Second, the autonomic nervous system—specifically its sympathetic and parasympathetic branches—provides a rapid, bidirectional communication channel (Mayer et al., 2015). Third, the enteric nervous system, an extensive neural network within the gut wall often called the “second brain,” can orchestrate local digestive functions independently (Furness, 2012). Fourth, the gut microbiota, a vast community of intestinal microorganisms, generates numerous bioactive compounds through metabolic activity (He et al., 2024). These components communicate continuously through neural, endocrine, and immune signaling pathways, forming a complex, dynamic network (Figure 1).

### 2.2 Key neural pathways of gut-brain axis

Direct neural connections are fundamental to the gut-brain axis's bidirectional communication. The vagus nerve is the principal parasympathetic pathway, transmitting the majority of sensory information from the gut to the brain and carrying regulatory commands back to the viscera (Bonaz et al., 2018). In contrast, spinal-sympathetic pathways are chiefly activated during stress, suppressing gastrointestinal motility and blood flow (Teff, 2008). Visceral afferent nerve fibers, distributed throughout the gut, detect local mechanical, chemical, and noxious stimuli and relay this sensory input to the central nervous system via the spinal cord (Furness, 2012; Yu et al., 2020). Together, these neural circuits enable the brain to monitor gut status in real time and modulate its function accordingly.

### 2.3 Humoral and Immune Mediators

Signaling within the gut-brain axis also depends heavily on circulating biochemical and immune mediators. Pro-inflammatory and anti-inflammatory cytokines can, during systemic inflammation, cross the blood-brain barrier or signal via nerves to influence central nervous system activity, thereby affecting mood and cognition (Zhang H. et al., 2025). Neuropeptides—such as gut-derived peptide YY and glucagon-like peptide-1, along with brain-derived corticotropin-releasing hormone—coordinate appetite, stress responses, and gut function (Holzer and Farzi, 2014). Additionally, short-chain fatty acids, produced by gut bacteria fermenting dietary fiber, serve as crucial microbial messengers that influence host immunity and neurology both locally and systemically after entering circulation (Silva et al., 2020).

### 2.4 Surgical and stress-induced perturbation of the gut-brain axis

Surgery and the associated perioperative stress represent a potent disruptor of gut-brain axis homeostasis (Danehower, 2021). The trauma of surgery initiates a systemic inflammatory response, characterized by the release of cytokines that can disrupt central nervous system function (Danehower, 2021). Concurrently, neuroendocrine stress responses and reduced splanchnic perfusion can compromise the intestinal epithelial barrier, increasing its permeability (Morys et al., 2024). This “leaky gut” may allow bacterial products to translocate into the bloodstream, potentially exacerbating systemic inflammation (Lange et al., 2025). Furthermore, factors like opioids, fasting, and antibiotics can induce a rapid shift in microbial community structure and function, known as dysbiosis (Klingensmith and Coopersmith, 2016). These three perturbations—systematic inflammation, intestinal barrier dysfunction, and gut dysbiosis—frequently interact in a synergistic and often self-perpetuating manner, establishing a pathophysiological vicious cycle. Clinically, this cycle manifests directly as postoperative gastrointestinal dysfunction, encompassing conditions such as ileus, feeding intolerance, and delayed bowel recovery (Mazzotta et al., 2020). Such complications not only prolong hospitalization but also perpetuate systemic inflammation and critically hinder functional recovery, particularly following major abdominal or colorectal procedures (Harnsberger et al., 2019; Bain et al., 2023). Collectively, these findings underscore the pivotal role of gastrointestinal physiology and gut-brain axis integrity in determining overall surgical outcomes, linking molecular and systemic perturbations to concrete clinical morbidity (Lubbers et al., 2010).

## 3 Potential regulatory mechanisms of nerve block on the gut-brain axis

**Figure 2:**
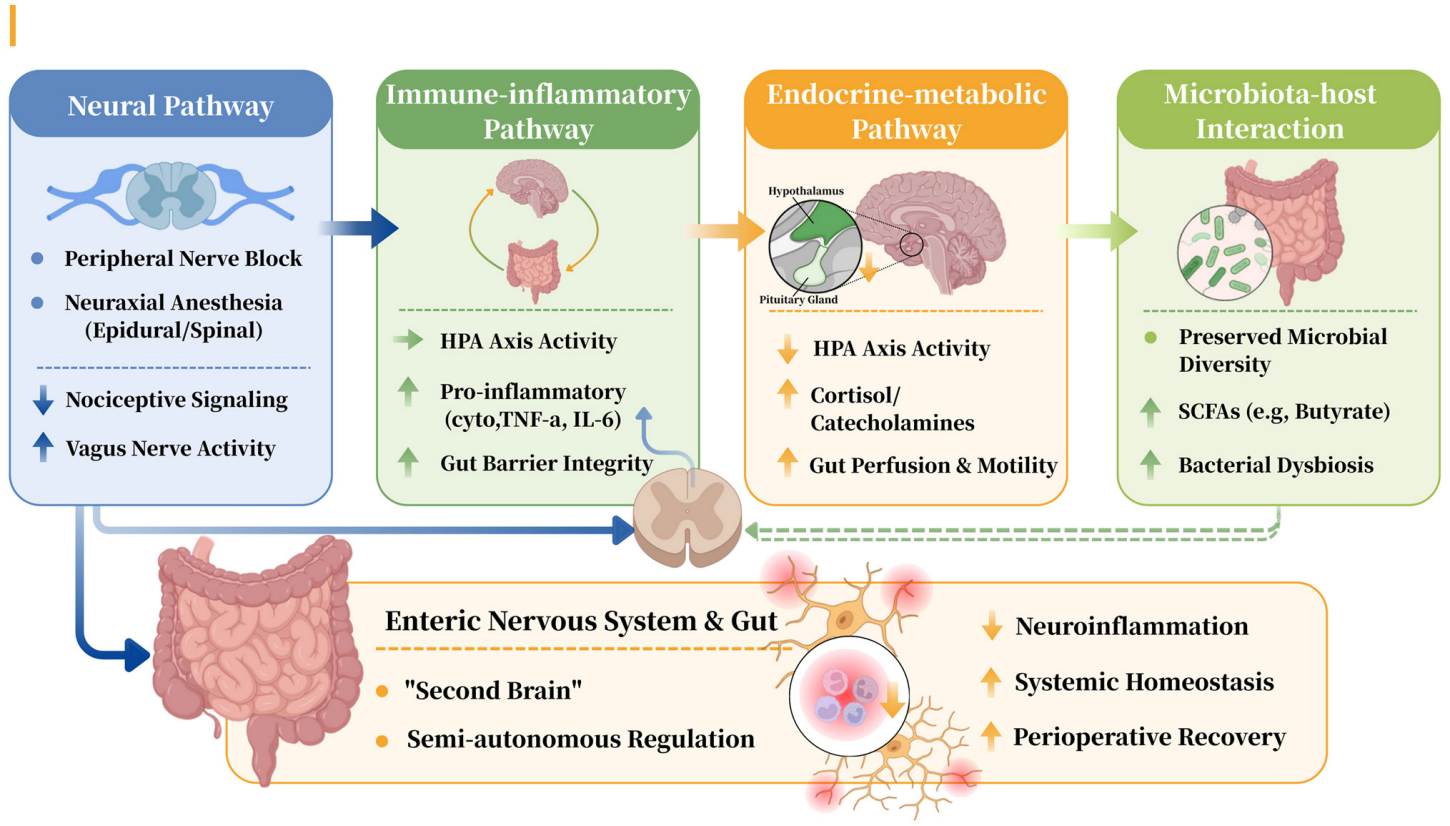
Multi-pathway modulation of the gut-brain axis by regional anesthesia.

The systemic benefits of regional anesthesia may arise primarily from its multi-faceted modulation of the integrated gut-brain axis (Figure 2). Importantly, we do not attribute all systemic effects of regional anesthesia to gut-brain axis pathways. Instead, we hypothesize that some of its systemic benefits may involve—directly or indirectly—gut-brain interactions, operating in conjunction with broader autonomic and neuroimmune mechanisms.

However, regional anesthesia encompasses a diverse range of techniques that vary in their anatomical targets, duration, and neural specificity, such as neuraxial versus peripheral nerve blocks (Axelsson and Gupta, 2009; Jeng et al., 2010). This inherent variability complicates the attribution of specific physiological effects—particularly those involving systemic or gut-brain pathways—to a singular mechanism (Reysner et al., 2024). Consequently, extrapolating findings across different techniques requires careful and contextual interpretation.

While regional anesthesia broadly suppresses sympathetic activity and enhances vagal tone, only part of this autonomic shift directly engages enteric neural pathways (Introna et al., 1995). The specific role of the enteric nervous system (ENS) in these processes remains incompletely understood, with current evidence supporting its plausible, rather than definitive, involvement (Heiss and Olofsson, 2019). Beyond its role within autonomic pathways, the ENS may also perform distinct, locally autonomous regulatory functions independent of vagal signaling (Fung and Vanden Berghe, 2020). Studies suggest that the ENS modulates enteric reflexes governing peristalsis and secretion, interacts bidirectionally with intestinal macrophages in the lamina propria, and influences epithelial barrier function via enteric glial cells (Spencer and Hu, 2020; Meroni et al., 2019). Additionally, ENS regulation of enteroendocrine signaling may alter local hormone release, potentially affecting systemic neuroendocrine and immune responses (Vergnolle and Cirillo, 2018; Latorre et al., 2016). Although direct evidence linking these ENS-specific mechanisms to the systemic effects of regional anesthesia remains limited, their functional and anatomical independence supports their plausible contribution within the broader gut-brain axis. Similarly, influences from the microbiota are more appropriately viewed as secondary or permissive modulators within this regulatory network (Geng et al., 2022). Advances in local anesthetic formulations have further reinforced the clinical value of these systemic benefits by optimizing pharmacodynamic profiles (Skolnik and Gan, 2014). For instance, liposome-encapsulated bupivacaine offers extended postoperative analgesia through sustained drug release, enabling prolonged modulation of these pathways (Hu et al., 2013; Gorfine et al., 2011; Hamilton et al., 2022). Meanwhile, the growing preference for ropivacaine leverages its lower cardiotoxicity and milder motor block to support earlier and safer postoperative mobilization and rehabilitation (Mather and Chang, 2001; Tai et al., 2022). The underlying mechanisms of gut-brain axis modulation can be organized into four interconnected categories.

### 3.1 Neural pathway intervention

Nerve blockade exerts its most direct effect through physical interruption of neural signaling pathways. Firstly, by blocking nociceptive afferent signals from the surgical site, it significantly dampens the activation of the hypothalamic-pituitary-adrenal (HPA) axis, thereby reducing the systemic neuroendocrine stress response at its source (Abram, 2000). Secondly, regional anesthesia, especially neuraxial blocks, modulates the autonomic nervous system balance. It inhibits sympathetic outflow while potentially promoting a relative dominance of vagal (parasympathetic) tone (Li et al., 2003). This shift toward parasympathetic predominance favors gastrointestinal motility, improves gut mucosal blood flow, and promotes an anti-inflammatory systemic environment (Tracey, 2002; de Jonge et al., 2005). The autonomic influence on gut function is likely mediated via direct modulation of enteric neural circuits, although the precise signaling integration within the ENS warrants further elucidation.

### 3.2 Immune and inflammatory modulation

Through these neural effects, nerve blockade induces significant immunomodulatory changes. Locally, by limiting nociceptive signaling and sympathetic activity, it suppresses the release of pro-inflammatory cytokines (e.g., tumor necrosis factor-alpha, interleukin-6) from the surgical site (Occhinegro et al., 2023). Systemically, the attenuated stress response and reduced sympathetic tone help preserve intestinal immune homeostasis (Populin et al., 2021; Mallesh et al., 2022). This may involve a shift toward an anti-inflammatory phenotype in gut-resident immune cells and helps maintain the integrity of the intestinal mucosal barrier, reducing bacterial translocation and subsequent systemic inflammation (Populin et al., 2021; Mallesh et al., 2022).

### 3.3 Endocrine and metabolic effects

Nerve blockade also has bidirectional effects on the endocrine system. By suppressing HPA axis activation, it directly lowers circulating levels of stress hormones like cortisol and catecholamines (Carli et al., 2002). Normalizing these hormone levels alleviates associated metabolic disturbances and removes their suppressive effects on gut function and immunity (Carli et al., 2002). Furthermore, the improved autonomic balance may positively influence enteroendocrine cell function (Furness et al., 2013). This could modulate the secretion of gut hormones such as ghrelin and glucagon-like peptide-1, thereby impacting broader metabolic homeostasis and nutrient signaling (Holst, 2007).

### 3.4 Indirect modulation of microbiota-host interaction

Finally, nerve blockade can indirectly influence the gut microbiota by altering the intestinal environment. Enhancing intestinal motility and perfusion supports a favorable niche for commensal bacteria, helping to maintain a stable and diverse microbial community (Huang et al., 2022; Xie et al., 2025). Concurrently, the neuro-immunologically mediated anti-inflammatory state may alter, the functional output of the microbiota (Xie et al., 2025). A key example is the potential modulation in the production of microbially derived metabolites, particularly short-chain fatty acids like butyrate, which have recognized anti-inflammatory and neuroactive properties (Bruning et al., 2020; Pang et al., 2026). This establishes a feedback loop where microbial metabolites further contribute to systemic physiological regulation. The microbiota likely functions as a responsive consortium within this system, whose composition and metabolic activity can be modulated by, and in turn modulate, host physiology altered by nerve blockade.

In summary, regional anesthesia acts as a powerful multi-system modulator rather than a purely local analgesic. By simultaneously targeting neural, immune, endocrine, and microbial communication pathways, it helps restore perioperative gut-brain axis homeostasis. This integrative mechanism underpins its potential to provide systemic protective effects beyond pain control.

## 4 Evidence synthesis: insights from preclinical and clinical research

The integration of evidence from preclinical and clinical studies is crucial for understanding the gut-brain axis effects of regional anesthesia. This synthesis supports the proposed systemic mechanisms and highlights current knowledge gaps (Supplementary Table 1).

### 4.1 Animal studies

Animal models offer valuable insights into the direct mechanisms of nerve blocks (Wu et al., 2022). Studies in abdominal surgery models show that neuraxial or regional blocks, compared to general anesthesia, significantly improve postoperative gastrointestinal recovery and reduce ileus (Boeckxstaens and Jonge, 2009; De Winter, 2003). These benefits appear linked to the modulation of neuroinflammation, such as reduced microglial activation in the brain (Wu et al., 2022; Zou et al., 2024). Furthermore, nerve blockade in animals helps preserve a healthier gut microbial balance after surgery, preventing severe dysbiosis and maintaining levels of beneficial metabolites like butyrate (Zou et al., 2024; Glynn et al., 2025). These findings provide experimental support for the interconnected neural, immune, and microbial pathways proposed in the gut-brain axis hypothesis.

Critically, although direct causal evidence is still limited, evidence from animal models converges with other lines of inquiry to suggest that the ENS may function as a key mediator in the gut-brain axis (Sharkey and Mawe, 2023). Current evidence supports a dual role for the ENS. First, it acts as a downstream responder, influenced by reduced sympathetic activity and attenuated inflammation (Progatzky and Pachnis, 2022; Progatzky et al., 2021). Second, it may actively regulate immune signaling, enteroendocrine pathways, and vagal afferent activity (Kaelberer et al., 2018). The ENS is anatomically and functionally situated to integrate neural, immune, and endocrine signals (Sharkey and Mawe, 2023). Within this framework, through these mechanisms, the ENS can amplify and integrate central nervous system effects, which helps explain how regional anesthesia may produce systemic benefits via the gut-brain axis (Matteoli et al., 2014).

### 4.2 Clinical observations

Clinical research consistently shows associations between regional anesthesia and improved patient outcomes. Its use is correlated with a lower incidence of postoperative delirium, potentially due to reduced systemic inflammation and opioid use (Zhuang et al., 2022). Regional anesthesia is also associated with decreased risks of surgical site infections and pulmonary complications, suggesting a role in supporting immune function (Lee et al., 2011). Additionally, patients often experience faster return of bowel function, shorter hospital stays, and improved early mobility when regional techniques are employed (Zhuang et al., 2022; Levy et al., 2011). These clinical correlations align with the systemic benefits suggested by animal research and the gut-brain axis framework. Notably, perioperative clinical studies indicate that regional anesthesia has been consistently shown to modulate autonomic balance, inflammatory tone, stress signaling, and gastrointestinal function—processes in which the ENS is positioned to play an integrative role (Reysner et al., 2024; Li et al., 2003). Collectively, available clinical evidence suggests an indirect link between regional anesthesia and gut-brain axis physiology. However, this does not establish a central or required mediating role, underscoring the exploratory nature of the present perspective.

### 4.3 Evidence from veterinary medicine

In addition to experimental models and human clinical studies, translational insights can also be drawn from veterinary medicine, where regional anesthesia has been widely practiced in diverse animal species. Regional anesthesia holds a long-established role in veterinary medicine and provides valuable translational insights (Edmondson, 2008). In large animals such as cattle and horses, surgical procedures—especially gastrointestinal and orthopedic interventions—are routinely performed under locoregional anesthesia alone (Román Durá et al., 2025). This preference stems from the increased risks of general anesthesia in these species. Standing abdominal procedures under nerve blocks, for instance, are particularly common in bovine practice (D'Anselme et al., 2022).

For small animals and equine surgery, regional anesthesia is a key element of multimodal analgesic strategies, supporting opioid-sparing approaches (Grubb and Lobprise, 2020). Its growing adoption has been accelerated in recent years by reduced opioid availability related to the human opioid crisis (Kogan et al., 2019). These veterinary applications highlight both the systemic relevance and practical utility of regional anesthesia across species, reinforcing its reconceptualization as a broadly applicable physiological modulator (Clarke et al., 2019). This body of veterinary research further contributes to the converging evidence suggesting a potential mediating role for the ENS, as the modulation of autonomic and gastrointestinal functions observed clinically is also a consistent feature of regional anesthesia in animal species.

### 4.4 Limitations

It is important to critically acknowledge the limitations of the current evidence base. Most clinical data demonstrate association, not proven causation; benefits may be confounded by reduced opioid use and other perioperative factors (Tanios et al., 2025). Crucially, direct evidence in humans linking nerve blocks to specific gut-brain axis pathways remains largely absent (Minerbi and Shen, 2022). There is a significant lack of studies that track the complete mechanistic cascade from neural blockade to measurable changes in human immune markers, hormones, and gut metabolites (Ekatodramis, 2001). Therefore, while the hypothesis is well-supported and plausible, more direct translational research is needed to establish definitive causal mechanisms in patients.

## 5 Translational prospects

Moving beyond its traditional role as a local analgesic, regional anesthesia should be recognized as a perioperative intervention with significant potential for systemic modulation via the gut-brain axis. This reconceptualization opens new avenues for translating this understanding into clinical practice and research.

### 5.1 Optimization of perioperative application

The clinical application of regional anesthesia should be integrated into broader perioperative strategies, such as Enhanced Recovery after Surgery protocols (Mancel et al., 2021; Campoy, 2022). Its value should be assessed not only for pain relief but also for its potential to improve gastrointestinal recovery, modulate immune function, and provide neuroprotection (Zhang T. et al., 2025; Bosenberg and Flick, 2013). Anesthesia planning should therefore consider the systemic, gut-brain-mediated benefits of nerve blocks, shifting the focus from isolated analgesia to comprehensive rehabilitation support (Bosenberg and Flick, 2013).

### 5.2 Potential role in chronic disease management

Given the centrality of the gut-brain axis in chronic conditions, regional anesthesia may have therapeutic relevance beyond the operating room. For example, targeted nerve blocks could help manage refractory visceral pain in disorders like irritable bowel syndrome by modulating aberrant gut-brain signaling (Mayer et al., 2015). Furthermore, its modulatory effects on inflammation and metabolism suggest a potential adjuvant role in metabolic surgery perioperative care to improve long-term outcomes (Deer et al., 2014; Aron-Wisnewsky and Clément, 2016). This indicates a possible expansion of its utility into the management of select chronic diseases.

### 5.3 Personalized medicine

A personalized approach could optimize nerve block therapy by accounting for individual differences in gut-brain axis physiology. Preoperative profiling using biomarkers related to microbiota, inflammation, or stress response might help identify patients most likely to benefit from specific regional techniques (Lee et al., 2025; Pearse et al., 2011). This would enable a shift from standardized protocols to tailored strategies that select the optimal type, timing, and duration of blockade based on a patient's unique physiological profile (De Hert et al., 2011).

### 5.4 Future research directions

Future research should prioritize discovering and validating practical biomarkers to objectively assess gut-brain axis status before and after regional anesthesia (Dalile et al., 2019). Ideal biomarkers would reliably indicate key aspects such as intestinal permeability, systemic inflammation, or microbial metabolite levels. From a translational perspective, this aligns with a stepwise, testable framework wherein regional nerve blockade first alters autonomic and ENS activity, subsequently driving downstream immune, endocrine, and gut barrier responses to produce systemic clinical effects (Reysner et al., 2024; Schäper et al., 2013; Mahajan et al., 2017; Tracey, 2007). Accordingly, biomarker discovery should span these stages and include autonomic measures (e.g., heart rate variability), inflammatory markers, gut permeability indicators, and enteroendocrine hormones (Wells et al., 2017).

Rigorous, mechanistic clinical trials are needed to establish causality between nerve blocks and specific gut-brain axis outcomes. Future mechanistic studies should directly validate these proposed physiological pathways and clarify causal relationships beyond mere anatomical or physiological overlap. These studies should measure intermediate endpoints like neuroendocrine or inflammatory markers to directly test the proposed pathways (Evered et al., 2018).

Notably, a key limitation in current evidence is the lack of experimental studies that investigate the causal role of the ENS in mediating the systemic effects of regional anesthesia (Langness et al., 2017). Animal models employing chemical or genetic ENS ablation could offer a direct means to test whether—and to what extent—ENS integrity is necessary for regional anesthesia to modulate inflammation, endocrine signaling, and gut-brain communication (Grubišić et al., 2022; Yoneda et al., 2002). Addressing this gap would enhance mechanistic understanding and help differentiate ENS-mediated effects from those primarily driven by central autonomic pathways.

Despite its potential advantages, the broader implementation of regional anesthesia in such research and subsequent clinical translation faces significant challenges. Its successful application necessitates thorough anatomical knowledge, technical skill, and experience in ultrasound-guided procedures (Niazi et al., 2012). However, current variations in training quality and procedural exposure across anesthesiology programs can hinder consistent adoption and the reliable application required for high-quality study protocols and eventual widespread clinical use (Ardon et al., 2023). To overcome these barriers, parallel efforts are essential to develop standardized curricula, implement simulation-based training, and establish competency-based certification, thereby ensuring both the safety and methodological rigor necessary for its effective investigation and application (Sites et al., 2010; Udani et al., 2015).

Expanding on this, research should investigate whether regional anesthesia works synergistically with other gut-brain-targeted therapies, such as specific probiotics, dietary regimens, or neuromodulation techniques (Cryan et al., 2019). Such multimodal strategies may offer superior outcomes in modulating perioperative physiology and enhancing recovery.

## 6 Summary

This perspective has argued that regional anesthesia exerts effects far beyond local pain control by modulating the gut-brain axis. Based on the presented evidence, we conclude with the following key points and a call to action. Regional anesthesia should be conceptualized as a systemic modulator, influencing physiology through neural, immune, endocrine, and microbial pathways linked to the gut-brain axis. This broader understanding elevates its role from a perioperative analgesic to a potential cornerstone of holistic recovery strategies.

In practice, clinicians should consider nerve blocks as a key intervention for regulating the body's integrated stress and recovery responses to surgery. Utilizing this multi-system perspective can optimize patient management, aiming not only for analgesia but also for improved gastrointestinal function, reduced inflammation, and enhanced overall recovery.

Advancing this field requires dedicated collaboration across multiple disciplines. Experts in anesthesiology, gastroenterology, neuroscience, microbiology, and surgery should work together to design targeted clinical trials and develop practical monitoring tools. This collaborative effort is essential to translate the gut-brain axis paradigm into effective, personalized patient care and to foster a more integrative model of perioperative medicine.

## Data Availability

The original contributions presented in the study are included in the article/Supplementary material, further inquiries can be directed to the corresponding author.

## References

[B1] Abram S. E. (2000). Neural blockade for neuropathic pain. Clin. J. Pain. 16, S56–61. doi: 10.1097/00002508-200006001-00010 10870741

[B2] Ardon A. Bojaxhi E. Clendenen S. McClain R. Gillespie N. Robards C. . (2023). A change in the educational landscape of regional anesthesia: a national survey of anesthesiology residency programs to assess the correlation between the frequency of teaching a block technique and clinical importance. Cureus. 15:e37869. doi: 10.7759/cureus.37869 37223208 PMC10202666

[B3] Aron-Wisnewsky J. Clément K. (2016). The gut microbiome, diet, and links to cardiometabolic and chronic disorders. Nat. Rev. Nephrol. 12, 169–181. doi: 10.1038/nrneph.2015.191 26616538

[B4] Axelsson K. Gupta A. (2009). Local anaesthetic adjuvants: neuraxial versus peripheral nerve block. Curr. Opin. Anaesthesiol. 22, 649–654. doi: 10.1097/ACO.0b013e32832ee847 19593120

[B5] Bain C. R. Myles P. S. Martin C. Wallace S. Shulman M. A. Corcoran T. . (2023). Postoperative systemic inflammation after major abdominal surgery: patient-centred outcomes. Anaesthesia. 78, 1365–1375. doi: 10.1111/anae.16104 37531295 PMC10952313

[B6] Bingham A. E. Fu R. Horn J. L. Abrahams M. S. (2012). Continuous peripheral nerve block compared with single-injection peripheral nerve block: a systematic review and meta-analysis of randomized controlled trials. Reg. Anesth. Pain Med. 37, 583–594. doi: 10.1097/AAP.0b013e31826c351b 23080349

[B7] Boeckxstaens G. E. Jonge d. e. (2009). WJ. Neuroimmune mechanisms in postoperative ileus. Gut. 58, 1300–1311. doi: 10.1136/gut.2008.169250 19671558

[B8] Bonaz B. Bazin T. Pellissier S. (2018). The vagus nerve at the interface of the microbiota-gut-brain axis. Front. Neurosci 12:49. doi: 10.3389/fnins.2018.00049 29467611 PMC5808284

[B9] Bosenberg A. Flick R. P. (2013). Regional anesthesia in neonates and infants. Clin. Perinatol. 40, 525–538. doi: 10.1016/j.clp.2013.05.011 23972755

[B10] Bruning J. Chapp A. Kaurala G. A. Wang R. Techtmann S. Chen Q. H. . (2020). Gut microbiota and short chain fatty acids: influence on the autonomic nervous system. Neurosci. Bull. 36, 91–95. doi: 10.1007/s12264-019-00410-8 31301036 PMC6940411

[B11] Campoy L. (2022). Development of enhanced recovery after surgery (ERAS) protocols in veterinary medicine through a one-health approach: the role of anesthesia and locoregional techniques. J. Am. Vet. Med. Assoc. 260, 1751–1759. doi: 10.2460/javma.22.08.0354 36108100

[B12] Carabotti M. Scirocco A. Maselli M. A. Severi C. (2015). The gut-brain axis: interactions between enteric microbiota, central and enteric nervous systems. Ann Gastroenterol. 28, 203–209. 25830558 PMC4367209

[B13] Carli F. Mayo N. Klubien K. Schricker T. Trudel J. Belliveau P. . (2002). Epidural analgesia enhances functional exercise capacity and health-related quality of life after colonic surgery: results of a randomized trial. Anesthesiology. 97, 540–549. doi: 10.1097/00000542-200209000-00005 12218518

[B14] Clarke D. L. Drobatz K. J. Korzekwa C. Nelson L. S. Perrone J. (2019). Trends in opioid prescribing and dispensing by veterinarians in Pennsylvania. JAMA Netw. Open. 2:e186950. doi: 10.1001/jamanetworkopen.2018.6950 30646207 PMC6484550

[B15] Costes L. M. van der Vliet J. van Bree S. H. Boeckxstaens G. E. Cailotto C. (2014). Endogenous vagal activation dampens intestinal inflammation independently of splenic innervation in postoperative ileus. Auton Neurosci. 185, 76–82. doi: 10.1016/j.autneu.2014.07.006 25103359

[B16] Cryan J. F. O'Riordan K. J. Cowan C. S. M. Sandhu K. V. Bastiaanssen T. F. S. Boehme M. . (2019). The microbiota-gut-brain axis. Physiol. Rev. 99, 1877–2013. doi: 10.1152/physrev.00018.2018 31460832

[B17] Dalile B. Van Oudenhove L. Vervliet B. Verbeke K. (2019). The role of short-chain fatty acids in microbiota-gut-brain communication. Nat. Rev. Gastroenterol. Hepatol. 16, 461–478. doi: 10.1038/s41575-019-0157-3 31123355

[B18] Danehower S. (2021). Targeting gut dysbiosis as a means to enhance recovery from surgical brain injury. Surg. Neurol. Int. 12:210. doi: 10.25259/SNI_72_2021 34084637 PMC8168676

[B19] D'Anselme O. Hartnack A. Andrade J. S. S. Alfaro Rojas C. Ringer S. K. de Carvalho Papa P. (2022). Description of an ultrasound-guided erector spinae plane block and comparison to a blind proximal paravertebral nerve block in cows: a cadaveric study. Animals 12:2191. doi: 10.3390/ani12172191 36077911 PMC9454813

[B20] De Hert S. Imberger G. Carlisle J. Diemunsch P. Fritsch G. Moppett I. . (2011). Preoperative evaluation of the adult patient undergoing non-cardiac surgery: guidelines from the European Society of Anaesthesiology. Eur. J. Anaesthesiol. 28, 684–722. doi: 10.1097/EJA.0b013e3283499e3b 21885981

[B21] de Jonge W. J. van der Zanden E. P. The F. O. Bijlsma M. F. van Westerloo D. J. Bennink R. J. . (2005). Stimulation of the vagus nerve attenuates macrophage activation by activating the Jak2-STAT3 signaling pathway. Nat. Immunol. 6, 844–851. doi: 10.1038/ni1229 16025117

[B22] De Winter B. Y. (2003). Study of the pathogenesis of paralytic ileus in animal models of experimentally induced postoperative and septic ileus. Verh K Acad Geneeskd Belg. 65, 293–324. 14671847

[B23] Deer T. R. Mekhail N. Provenzano D. Pope J. Krames E. Leong M. . (2014). The appropriate use of neurostimulation of the spinal cord and peripheral nervous system for the treatment of chronic pain and ischemic diseases: the Neuromodulation Appropriateness Consensus Committee. Neuromodulation 17, 515–550. doi: 10.1111/ner.12208 25112889

[B24] Dubowitz J. A. Sloan E. K. Riedel B. J. (2018). Implicating anaesthesia and the perioperative period in cancer recurrence and metastasis. Clin Exp Metastasis. 35, 347–358. doi: 10.1007/s10585-017-9862-x 28894976

[B25] Edmondson M. A. (2008). Local and regional anesthesia in cattle. Vet. Clin. North Am. Food Anim. Pract. 24, 211–226. doi: 10.1016/j.cvfa.2008.02.013 18471564

[B26] Ekatodramis G. (2001). Regional anesthesia and analgesia: their role in postoperative outcome. Curr. Top Med. Chem. 1, 183–192. doi: 10.2174/1568026013395236 11895134

[B27] Evered L. Silbert B. Knopman D. S. Scott D. A. DeKosky S. T. Rasmussen L. S. . (2018). Recommendations for the nomenclature of cognitive change associated with anaesthesia and surgery-2018. Br. J. Anaesth. 121, 1005–1012. doi: 10.1016/j.bja.2017.11.087 30336844 PMC7069032

[B28] Fung C. Vanden Berghe P. (2020). Functional circuits and signal processing in the enteric nervous system. Cell. Mol. Life Sci. 77, 4505–4522. doi: 10.1007/s00018-020-03543-6 32424438 PMC7599184

[B29] Furness J. B. (2012). The enteric nervous system and neurogastroenterology. Nat. Rev. Gastroenterol. Hepatol. 9, 286–294. doi: 10.1038/nrgastro.2012.32 22392290

[B30] Furness J. B. Rivera L. R. Cho H. J. Bravo D. M. Callaghan B. (2013). The gut as a sensory organ. Nat Rev Gastroenterol Hepatol. 10, 729–740. doi: 10.1038/nrgastro.2013.180 24061204

[B31] Geng Z. H. Zhu Y. Li Q. L. Zhao C. Zhou P. H. (2022). Enteric nervous system: the bridge between the gut microbiota and neurological disorders. Front. Aging Neurosci. 14, 810483. doi: 10.3389/fnagi.2022.810483 35517052 PMC9063565

[B32] Glynn J. M. Strohl J. J. Bagnall-Moreau C. Carrión J. Huerta P. T. (2025). Butyrate preserves entorhinal-hippocampal spatial coding and blood brain barrier integrity in mice with depleted gut microbiome. bioRxiv [Preprint]. 2025:666609. doi: 10.1101/2025.07.24.666609 40766709 PMC12324207

[B33] Gorfine S. R. Onel E. Patou G. Krivokapic Z. V. (2011). Bupivacaine extended-release liposome injection for prolonged postsurgical analgesia in patients undergoing hemorrhoidectomy: a multicenter, randomized, double-blind, placebo-controlled trial. Dis. Colon. Rectum. 54, 1552–1559. doi: 10.1097/DCR.0b013e318232d4c1 22067185

[B34] Graff V. Gabutti L. Treglia G. Pascale M. Anselmi L. Cafarotti S. . (2023). Perioperative costs of local or regional anesthesia versus general anesthesia in the outpatient setting: a systematic review of recent literature. Braz. J. Anesthesiol. 73, 316–339. doi: 10.1016/j.bjane.2021.09.012 34627828 PMC10240220

[B35] Grosu I. Lavand'homme P. (2015). Continuous regional anesthesia and inflammation: a new target. Minerva Anestesiol. 81, 1001–1009. 25317576

[B36] Grubb T. Lobprise H. (2020). Local and regional anaesthesia in dogs and cats: overview of concepts and drugs (Part 1). Vet. Med. Sci. 6, 209–217. doi: 10.1002/vms3.219 31965742 PMC7196681

[B37] Grubišić V. Bali V. Fried D. E. Eltzschig H. K. Robson S. C. Mazei-Robison M. S. . (2022). Enteric glial adenosine 2B receptor signaling mediates persistent epithelial barrier dysfunction following acute DSS colitis. Mucosal. Immunol. 15, 964–976. doi: 10.1038/s41385-022-00550-7 35869148 PMC9385475

[B38] Hahnenkamp K. Herroeder S. Hollmann M. W. (2004). Regional anaesthesia, local anaesthetics and the surgical stress response. Best Pract. Res. Clin. Anaesthesiol. 18, 509–527. doi: 10.1016/j.bpa.2004.01.004 15212342

[B39] Hamilton T. W. Knight R. Stokes J. R. Rombach I. Cooper C. Davies L. . (2022). Efficacy of liposomal bupivacaine and bupivacaine hydrochloride vs bupivacaine hydrochloride alone as a periarticular anesthetic for patients undergoing knee replacement: a randomized clinical trial. JAMA Surg. 157, 481–489. doi: 10.1001/jamasurg.2022.0713 35385072 PMC8988023

[B40] Harnsberger C. R. Maykel J. A. Alavi K. (2019). Postoperative Ileus. Clin. Colon. Rectal. Surg. 32, 166–170. doi: 10.1055/s-0038-1677003 31061645 PMC6494613

[B41] He Y. Wang K. Su N. Yuan C. Zhang N. Hu X. . (2024). Microbiota-gut-brain axis in health and neurological disease: interactions between gut microbiota and the nervous system. J. Cell Mol. Med. 28:e70099. doi: 10.1111/jcmm.70099 39300699 PMC11412916

[B42] Heiss C. N. Olofsson L. E. (2019). The role of the gut microbiota in development, function and disorders of the central nervous system and the enteric nervous system. J. Neuroendocrinol. 31:e12684. doi: 10.1111/jne.12684 30614568

[B43] Holst J. J. (2007). The physiology of glucagon-like peptide 1. Physiol Rev. 87, 1409–1039. doi: 10.1152/physrev.00034.2006 17928588

[B44] Holzer P. Farzi A. (2014). Neuropeptides and the microbiota-gut-brain axis. Adv. Exp. Med. Biol. 817, 195–219. doi: 10.1007/978-1-4939-0897-4_9 24997035 PMC4359909

[B45] Hu D. Onel E. Singla N. Kramer W. G. Hadzic A. (2013). Pharmacokinetic profile of liposome bupivacaine injection following a single administration at the surgical site. Clin. Drug. Investig. 33, 109–115. doi: 10.1007/s40261-012-0043-z 23229686

[B46] Huang H. Zhou L. Yu Y. Liu S. Xu H. Xu Z. . (2022). Comparison of deep and moderate neuromuscular blockade on intestinal mucosal barrier in laparoscopic gastrectomy: a prospective, randomized, double-blind clinical trial. Front. Med. 8:789597. doi: 10.3389/fmed.2021.789597 35186973 PMC8847255

[B47] Hutton M. Brull R. Macfarlane A. J. R. (2018). Regional anaesthesia and outcomes. BJA Educ. 18, 52–56. doi: 10.1016/j.bjae.2017.10.002 33456810 PMC7807931

[B48] Introna R. Yodlowski E. Pruett J. Montano N. Porta A. Crumrine R. . (1995). Sympathovagal effects of spinal anesthesia assessed by heart rate variability analysis. Anesth. Analg. 80, 315–321. doi: 10.1213/00000539-199502000-00019 7818119

[B49] Jeng C. L. Torrillo T. M. Rosenblatt M. A. (2010). Complications of peripheral nerve blocks. Br. J. Anaesth. 105, i97–107. doi: 10.1093/bja/aeq273 21148659

[B50] Kaelberer M. M. Buchanan K. L. Klein M. E. Barth B. B. Montoya M. M. Shen X. . (2018). A gut-brain neural circuit for nutrient sensory transduction. Science 361:eaat5236. doi: 10.1126/science.aat5236 30237325 PMC6417812

[B51] Klingensmith N. J. Coopersmith C. M. (2016). The gut as the motor of multiple organ dysfunction in critical illness. Crit Care Clin. 32, 203–212. doi: 10.1016/j.ccc.2015.11.004 27016162 PMC4808565

[B52] Kogan L. Hellyer P. Rishniw M. Schoenfeld-Tacher R. (2019). The US opioid epidemic and its impact on US general practice veterinarians. Fron.t Vet. Sci. 6:222. doi: 10.3389/fvets.2019.00222 31334257 PMC6620788

[B53] Lange U. G. Lehr K. Thieme R. Hoffmeister A. Feisthammel J. Gockel I. . (2025). The influence of antibiotic and mechanical bowel preparation on the microbiome in colorectal cancer surgery: a pilot study. Surg. Pract. Sci. 22:100302. doi: 10.1016/j.sipas.2025.100302 40838261 PMC12362692

[B54] Langness S. Kojima M. Coimbra R. Eliceiri B. P. Costantini T. W. (2017). Enteric glia cells are critical to limiting the intestinal inflammatory response after injury. Am. J. Physiol. Gastrointest. Liver Physiol. 312, G274–G282. doi: 10.1152/ajpgi.00371.2016 28082286

[B55] Latorre R. Sternini C. De Giorgio R. Greenwood-Van Meerveld B. (2016). Enteroendocrine cells: a review of their role in brain-gut communication. Neurogastroenterol Motil. 28, 620–630. doi: 10.1111/nmo.12754 26691223 PMC4842178

[B56] Lee H. K. Shin C. M. Chang Y. H. Jo H. Choi J. Choi Y. . (2025). Predictors of treatment response to fecal microbiota transplantation in irritable bowel syndrome: a pilot study. J. Neurogastroenterol. Motil. 31, 462–476. doi: 10.5056/jnm24183 41077748 PMC12527954

[B57] Lee J. S. Hayanga A. J. Kubus J. J. Makepeace H. Hutton M. Campbell D. A. . (2011). Local anesthesia: a strategy for reducing surgical site infections? World J. Surg. 35, 2596–602. doi: 10.1007/s00268-011-1298-x 21984145

[B58] Levy B. F. Scott M. J. Fawcett W. Fry C. Rockall T. A. (2011). Randomized clinical trial of epidural, spinal or patient-controlled analgesia for patients undergoing laparoscopic colorectal surgery. Br. J. Surg. 98, 1068–1078. doi: 10.1002/bjs.7545 21590762

[B59] Li Q. Guan L. Jiang J. (2003). Effect of general and epidural anesthesia on autonomic nervous system. Beijing Da Xue Xue Bao Yi Xue Ban. 35, 191–194. 12920842

[B60] Li T. Dong T. Cui Y. Meng X. Dai Z. (2022). Effect of regional anesthesia on the postoperative delirium: A systematic review and meta-analysis of randomized controlled trials. Front. Surg. 9:937293. doi: 10.3389/fsurg.2022.937293 35959124 PMC9360531

[B61] Liu S. S. Wu C. L. (2007). Effect of postoperative analgesia on major postoperative complications: a systematic update of the evidence. Anesth. Analg.104, 689–702. doi: 10.1213/01.ane.0000255040.71600.41 17312231

[B62] Lubbers T. Buurman W. Luyer M. (2010). Controlling postoperative ileus by vagal activation. World J. Gastroenterol. 16, 1683–1687. doi: 10.3748/wjg.v16.i14.1683 20379998 PMC2852814

[B63] Mahajan A. Takamiya T. Benharash P. Zhou W. (2017). Effect of thoracic epidural anesthesia on heart rate variability in a porcine model. Physiol. Rep. 5:e13116. doi: 10.14814/phy2.13116 28400498 PMC5392501

[B64] Mallesh S. Ten Hove A. S. Schneider R. Schneiker B. Efferz P. Kalff J. C. . (2022). Sympathetic innervation modulates mucosal immune homeostasis and epithelial host defense. Cells. 11:2606. doi: 10.3390/cells11162606 36010681 PMC9406312

[B65] Mancel L. Van Loon K. Lopez A. M. (2021). Role of regional anesthesia in enhanced recovery after surgery (ERAS) protocols. Curr. Opin. Anaesthesiol. 34, 616–625. doi: 10.1097/ACO.0000000000001048 34325463

[B66] Mather L. E. Chang D. H. (2001). Cardiotoxicity with modern local anaesthetics: is there a safer choice? Drugs. 61, 333–342. doi: 10.2165/00003495-200161030-00002 11293644

[B67] Matteoli G. Gomez-Pinilla P. J. Nemethova A. Di Giovangiulio M. Cailotto C. van Bree S. H. . (2014). A distinct vagal anti-inflammatory pathway modulates intestinal muscularis resident macrophages independent of the spleen. Gut. 63, 938–948. doi: 10.1136/gutjnl-2013-304676 23929694

[B68] Mayer E. A. Tillisch K. Gupta A. (2015). Gut/brain axis and the microbiota. J. Clin. Invest. 125, 926–938. doi: 10.1172/JCI76304 25689247 PMC4362231

[B69] Mazzotta E. Villalobos-Hernandez E. C. Fiorda-Diaz J. Harzman A. Christofi F. L. (2020). Postoperative ileus and postoperative gastrointestinal tract dysfunction: pathogenic mechanisms and novel treatment strategies beyond colorectal enhanced recovery after surgery protocols. Front. Pharmacol. 11:583422. doi: 10.3389/fphar.2020.583422 33390950 PMC7774512

[B70] Meroni E. Stakenborg N. Viola M. F. Boeckxstaens G. E. (2019). Intestinal macrophages and their interaction with the enteric nervous system in health and inflammatory bowel disease. Acta Physiol. 225:e13163. doi: 10.1111/apha.13163 29998613 PMC6519157

[B71] Minerbi A. Shen S. (2022). Gut microbiome in anesthesiology and pain medicine. Anesthesiology 137, 93–108. doi: 10.1097/ALN.0000000000004204 35486831 PMC9183187

[B72] Morys J. Małecki A. Nowacka-Chmielewska M. (2024). Stress and the gut-brain axis: an inflammatory perspective. Front. Mol. Neurosci. 17:1415567. doi: 10.3389/fnmol.2024.1415567 39092201 PMC11292226

[B73] Myles P. S. Peyton P. Silbert B. Hunt J. Rigg J. R. Sessler D. I. . (2011). Perioperative epidural analgesia for major abdominal surgery for cancer and recurrence-free survival: randomised trial. BMJ. 342, d1491–d1491. doi: 10.1136/bmj.d1491 21447587

[B74] Niazi A. U. Haldipur N. Prasad A. G. Chan V. W. (2012). Ultrasound-guided regional anesthesia performance in the early learning period: effect of simulation training. Reg. Anesth. Pain Med. 37, 51–54. doi: 10.1097/AAP.0b013e31823dc340 22179300

[B75] Occhinegro A. McAllen R. M. McKinley M. J. Martelli D. (2023). Acute inhibition of inflammation mediated by sympathetic nerves: the inflammatory reflex. Neuroimmunomodulation 30, 135–142. doi: 10.1159/000531469 37302390 PMC10428141

[B76] Pang S. Ren Z. Ding H. Chan P. (2026). Short-chain fatty acids mediate enteric and central nervous system homeostasis in Parkinson's disease: innovative therapies and their translation. Neural. Regen. Res. 21, 938–956. doi: 10.4103/NRR.NRR-D-24-01265 40313087 PMC12296502

[B77] Pearse R. M. Holt P. J. Grocott M. P. (2011). Managing perioperative risk in patients undergoing elective non-cardiac surgery. BMJ 343:d5759. doi: 10.1136/bmj.d5759 21976704

[B78] Populin L. Stebbing M. J. Furness J. B. (2021). Neuronal regulation of the gut immune system and neuromodulation for treating inflammatory bowel disease. FASEB Bioadv. 3, 953–966. doi: 10.1096/fba.2021-00070 34761177 PMC8565205

[B79] Progatzky F. Pachnis V. (2022). The role of enteric glia in intestinal immunity. Curr. Opin. Immunol. 77:102183. doi: 10.1016/j.coi.2022.102183 35533467 PMC9586875

[B80] Progatzky F. Shapiro M. Chng S. H. Garcia-Cassani B. Classon C. H. Sevgi S. . (2021). Regulation of intestinal immunity and tissue repair by enteric glia. Nature. 599, 125–130. doi: 10.1038/s41586-021-04006-z 34671159 PMC7612231

[B81] Reysner T. Wieczorowska-Tobis K. Kowalski G. Grochowicka M. Pyszczorska M. Mularski A. . (2024). The influence of regional anesthesia on the systemic stress response. Reports (MDPI). 7:89. doi: 10.3390/reports7040089 40757696 PMC12199975

[B82] Román Durá B. Dunham O. Grulke S. Salciccia A. Dupont J. Sandersen C. A. . (2025). Retrospective study on pre- and intraoperative predictors on the recovery quality of horses after general anesthesia. Vet. Sci. 12:262. doi: 10.3390/vetsci12030262 40266986 PMC11945850

[B83] Schäper J. Wagner A. Enigk F. Brell B. Mousa S. A. Habazettl H. . (2013). Regional sympathetic blockade attenuates activation of intestinal macrophages and reduces gut barrier failure. Anesthesiology 118, 134–142. doi: 10.1097/ALN.0b013e3182784c93 23221864

[B84] Schug S. A. Saunders D. Kurowski I. Paech M. J. (2006). Neuraxial drug administration: a review of treatment options for anaesthesia and analgesia. CNS Drugs. 20, 917–933. doi: 10.2165/00023210-200620110-00005 17044729

[B85] Sessler D. I. Pei L. Huang Y. Fleischmann E. Marhofer P. Kurz A. . (2019). Recurrence of breast cancer after regional or general anaesthesia: a randomised controlled trial. Lancet 394, 1807–1815. doi: 10.1016/S0140-6736(19)32313-X 31645288

[B86] Sharkey K. A. Mawe G. M. (2023). The enteric nervous system. Physiol. Rev. 103, 1487–1564. doi: 10.1152/physrev.00018.2022 36521049 PMC9970663

[B87] Shi W. Z. Miao Y. L. Yakoob M. Y. Cao J. B. Zhang H. Jiang Y. G. . (2014). Recovery of gastrointestinal function with thoracic epidural vs systemic analgesia following gastrointestinal surgery. Acta Anaesthesiol. Scandinavica 58, 923–932. doi: 10.1111/aas.12375 25060245

[B88] Silva Y. P. Bernardi A. Frozza R. L. (2020). The role of short-chain fatty acids from gut microbiota in gut-brain communication. Front. Endocrinol. 11:25. doi: 10.3389/fendo.2020.00025 32082260 PMC7005631

[B89] Sites B. D. Chan V. W. Neal J. M. Weller R. Grau T. Koscielniak-Nielsen Z. J. . (2010). The American society of regional anesthesia and pain medicine and the european society of regional anaesthesia and pain therapy joint committee recommendations for education and training in ultrasound-guided regional anesthesia. Reg. Anesth. Pain Med. 35, S74–S80. doi: 10.1097/AAP.0b013e3181d34ff5 20216029

[B90] Skolnik A. Gan T. J. (2014). New formulations of bupivacaine for the treatment of postoperative pain: liposomal bupivacaine and SABER-Bupivacaine. Expert. Opin. Pharmacother. 15, 1535–1542. doi: 10.1517/14656566.2014.930436 24992382

[B91] Spencer N. J. Hu H. (2020). Enteric nervous system: sensory transduction, neural circuits and gastrointestinal motility. Nat. Rev. Gastroenterol. Hepatol. 17, 338–351. doi: 10.1038/s41575-020-0271-2 32152479 PMC7474470

[B92] Tai Y. L. Peng L. Wang Y. Zhao Z. J. Li Y. N. Yin C. P. . (2022). Median effective concentration of ropivacaine for femoral nerve block maintaining motor function during knee arthroscopy in two age groups. J. Pain. Res. 15, 1647–1657. doi: 10.2147/JPR.S357750 35698568 PMC9188396

[B93] Tanios A. G. Gallagher E. L. McManus M. S. Riordan J. A. Harris I. A. Harvey L. A. . (2025). The effect of type of anaesthetic on delirium after surgery for acute hip fracture: an instrumental variable analysis to assess causation. Anaesth. Intensive Care. 53, 116–124. doi: 10.1177/0310057X241275116 39757848

[B94] Taylor A. McLeod G. (2020). Basic pharmacology of local anaesthetics. BJA Educ. 20, 34–41. doi: 10.1016/j.bjae.2019.10.002 33456928 PMC7808030

[B95] Teff K. L. (2008). Visceral nerves: vagal and sympathetic innervation. JPEN J. Parenter Enteral. Nutr. 32, 569–571. doi: 10.1177/0148607108321705 18753395

[B96] Tracey K. J. (2002). The inflammatory reflex. Nature. 420, 853–859. doi: 10.1038/nature01321 12490958

[B97] Tracey K. J. (2007). Physiology and immunology of the cholinergic antiinflammatory pathway. J. Clin. Invest. 117, 289–96. doi: 10.1172/JCI30555 17273548 PMC1783813

[B98] Udani A. D. Kim T. E. Howard S. K. Mariano E. R. (2015). Simulation in teaching regional anesthesia: current perspectives. Local Reg. Anesth 8, 33–43 doi: 10.2147/LRA.S68223 26316812 PMC4540124

[B99] Vergnolle N. Cirillo C. (2018). Neurons and glia in the enteric nervous system and epithelial barrier function. Physiology. 33, 269–280. doi: 10.1152/physiol.00009.2018 29897300 PMC6088142

[B100] Wang X. Q. Li H. Li X. N. Yuan C. H. Zhao H. (2021). Gut-brain axis: possible role of gut microbiota in perioperative neurocognitive disorders. Front. Aging Neurosci. 13, 745774. doi: 10.3389/fnagi.2021.745774 35002672 PMC8727913

[B101] Wells J. M. Brummer R. J. Derrien M. MacDonald T. T. Troost F. Cani P. D. . (2017). Homeostasis of the gut barrier and potential biomarkers. Am. J. Physiol. Gastrointest Liver Physiol. 312, G171–G193. doi: 10.1152/ajpgi.00048.2015 27908847 PMC5440615

[B102] Wu C. C. Chang C. Y. Tzeng C. Y. Huang J. H. Hung C. J. Chen W. Y. . (2022). Preventive intrathecal injection of bupivacaine alleviated microglia activation and neuropathic pain in a rat model of chronic constriction injury. Int. J. Mol. Sci. 23:7197. doi: 10.3390/ijms23137197 35806200 PMC9266705

[B103] Xie F. Du Q. Wang Q. Lu W. Song S. Yu J. . (2025). Lidocaine intervention attenuated postoperative delirium by reshaping intestinal flora and modulating microglia M2 type polarization and blood-brain barrier. Biochem. Biophys. Res. Commun. 778:152301. doi: 10.1016/j.bbrc.2025.152301 40684533

[B104] Yoneda A. Shima H. Nemeth L. Oue T. Puri P. (2002). Selective chemical ablation of the enteric plexus in mice. Pediatr. Surg. Int. 18, 234–237. doi: 10.1007/s003830100681 12021968

[B105] Yu C. D. Xu Q. J. Chang R. B. (2020). Vagal sensory neurons and gut-brain signaling. Curr. Opin. Neurobiol. 62, 133–140. doi: 10.1016/j.conb.2020.03.006 32380360 PMC7560965

[B106] Zhang H. Luan J. He L. Pan X. Zhang H. Li Y. . (2025). Role of the gut-brain axis in neurological diseases: molecular connections and therapeutic implications. Int. J. Mol. Med. 56:192. doi: 10.3892/ijmm.2025.5633 40937571 PMC12440273

[B107] Zhang T. Chung W. Orser B. A. (2025). Revisiting anesthesia-induced preconditioning for neuroprotection in the aging brain: a narrative review. Korean J. Anesthesiol. 78, 187–198. doi: 10.4097/kja.25073 40112780 PMC12142492

[B108] Zhuang X. He Y. Liu Y. Li J. Ma W. (2022). The effects of anesthesia methods and anesthetics on postoperative delirium in the elderly patients: a systematic review and network meta-analysis. Front. Aging Neurosci. 14:935716. doi: 10.3389/fnagi.2022.935716 36408115 PMC9670185

[B109] Zou Q. Han S. Liang J. Yan G. Wang Q. Wang Y. . (2024). Alleviating effect of vagus nerve cutting in Salmonella-induced gut infections and anxiety-like behavior via enhancing microbiota-derived GABA. Brain. Behav. Immun. 119, 607–620. doi: 10.1016/j.bbi.2024.04.034 38663772

