# Supplementary material for: Reconceptualizing regional anesthesia as a systemic modulator: a hypothesis-driven gut-brain axis perspective
Source: Front Neurosci. 2026 Mar 9;20:1785236. doi: 10.3389/fnins.2026.1785236 (PMC13006670; doi:10.3389/fnins.2026.1785236)
Supplement: Supplementary file 1 [file Table_1.docx]

**Supplementary Table 1. Evidence supporting gut–brain axis modulation by regional anesthesia**

| **Gut-brain axis pathway** | **Core mechanism** | **Key supporting evidence** | **Main limitations** |
| --- | --- | --- | --- |
| Neural pathway | Interruption of nociceptive afferent signaling attenuates HPA axis activation and shifts autonomic balance toward parasympathetic  dominance | Animal and clinical studies show reduced stress responses and improved postoperative gastrointestinal recovery with regional or neuraxial anesthesia | Human data are largely associative; direct measurements of autonomic and central neural activity are limited |
| Immune-  inflammatory pathway | Reduced sympathetic activity suppresses pro-inflammatory cytokine release and preserves  intestinal immune homeostasis and barrier integrity | Experimental studies demonstrate reduced intestinal inflammation and barrier dysfunction following sympathetic or regional blockade | Gut immune markers and permeability are rarely assessed in clinical trials; confounding perioperative factors exist |
| Endocrine-  metabolic pathway | Suppression of HPA axis lowers cortisol and catecholamine levels, improving systemic metabolic and immune milieu | Clinical studies associate regional anesthesia with attenuated stress hormone responses and improved functional recovery | Direct evidence linking nerve blocks to gut hormone modulation is scarce |
| Microbiota-  host interaction | Improved gut perfusion, motility, and immune balance stabilize microbial composition and enhance production of neuroactive metabolites (e.g., SCFAs) | Animal and emerging translational studies report mitigation of postoperative dysbiosis and preservation of beneficial metabolites | Causal evidence in humans remains limited; microbiota effects are indirect and influenced by multiple confounders |

**Abbreviations**: HPA, hypothalamic–pituitary–adrenal; SCFAs, short-chain fatty acids.
